# Should reporting of peri-neural invasion and extra prostatic extension be mandatory in prostate cancer biopsies? correlation with outcome in biopsy cases treated conservatively

**DOI:** 10.18632/oncotarget.24994

**Published:** 2018-04-17

**Authors:** Amar S. Ahmad, Vishnu Parameshwaran, Luis Beltran, Gabrielle Fisher, Bernard V. North, David Greenberg, Geraldine Soosay, Henrik Møller, Peter Scardino, Jack Cuzick, Daniel M. Berney, on behalf of the Transatlantic Prostate Group

**Affiliations:** ^1^ UK Center for Cancer Prevention, Wolfson Institute of Preventive Medicine, Queen Mary University of London, London, UK; ^2^ Department of Molecular Oncology, Barts Cancer Institute Queen Mary University of London, London, UK; ^3^ National Cancer Registration Service (Eastern Office), Public Health England, Cambridge, UK; ^4^ Department of Pathology, Queen’s Hospital, Romford, Essex, UK; ^5^ Cancer Epidemiology and Population Health, King's College London, London, UK; ^6^ Department of Urology, Memorial Sloan-Kettering Cancer Center, New York, NY, USA

**Keywords:** prostate cancer, perineural invasion, Gleason score, survival analysis

## Abstract

The identification of perineural invasion (PNI) and extraprostatic extension (ECE) in prostate cancer (PC) biopsies is time consuming and can be difficult. Although this is required information in many datasets, there is little evidence on their effect on outcome in patients treated conservatively. Cases of PC were identified from three cancer registries in the UK from men with clinically localized prostate cancer diagnosed by needle biopsy from 1990–2003. The endpoint was prostate cancer death (DOD). Patients treated radically within 6 months, those with objective evidence of metastases or who had prior hormone therapy were excluded. Follow-up was through cancer registries up until 2012. Deaths were divided into those from PC and those from other causes, according to WHO criteria. 988 biopsy cases (6522 biopsy cores) were centrally reviewed by three uropathologists and assigned a Gleason score and Grade Group (GG). The presence of both PNI and ECE was recorded. Of 988 patients, PNI was present in 288 (DOD = 75) and ECE in 23 (DOD = 5). On univariable analysis PNI was highly significantly associated with DOD (hazard ratio [HR] 2.28, 95% CI: 1.68, 3.1, log-rank test *p*-value = 4.8 × 10^–8^), but ECE was not (log-rank test *p*-value = 0.334). On multivariable analysis with GG, serum PSA (per 10%), clinical stage and extent of disease (per 10%), PNI lost significance (HR 1.16, 95% CI: 0.83, 1.63, likelihood ratio test *p*-value = 0.371). The utility of routinely examining prostate biopsies for ECE and PNI is doubtful as it is not independently associated with higher grade, stage or prognosis.

## INTRODUCTION

In spite of the fact that prostate cancer is the fourth most common cancer globally and the second most common cancer in men there remains uncertainty on the optimal management strategy for clinically localized tumors of low and intermediate grades. Increasing detection has led to many men who are more likely to die with their prostate cancer than of it. The identification of clinicopathological or other biomarkers which are risk factors for progression is therefore of great importance. Prostate cancer is diagnosed primarily by biopsy, and many factors have been used to predict the likelihood of progressive disease and need for radical therapy. Gleason score and recently the establishment of Grade Groups by the International Society of Urological Pathology [1] allows reasonable risk stratification, together with serum PSA, clinical stage and more recently imaging. Perineural invasion (PNI) by prostate cancer has long been considered a risk factor for disease progression.

Perineural invasion (PNI) is defined as “cancer tracking along or around a nerve within the perineural space”. Guidance from individual countries differs on whether PNI identification should be mandatory. While the Royal College of Pathologists requires reporting of PNI [2], the College of American Pathologists and International Collaboration on Cancer Reporting has suggested it is optional [3].

Many conflicting studies have been performed examining PNI in prostate cancer but the vast majority use either pathological surrogates or biochemical recurrence rather than prostatic cancer death as an outcome. None have been performed using prostate cancer death as an endpoint in patients with biopsies, who were initially treated in a conservative manner (Table 1).

**Table 1:** Death from prostate cancer in GG and PNI Groups

Grade Group	Alive/DOC			Death from Disease			Total
	PNI negative	PNI positive	Sum	PNI negative	PNI positive	Sum	
1	276	16	292	15	0	15	307
2	177	87	264	23	16	39	303
3	100	58	158	26	26	52	210
4	27	14	41	5	10	15	56
5	26	38	64	25	23	48	112
Sum	606	213	819	94	75	169	988

More rarely invasion of fat is seen in prostate biopsies. As fat is essentially absent from the prostate, any such case can be designated as extra prostatic extension (EPE), and TNM stage pT3 at least and may potentially assist in decisions for treatment. We wished to examine the hypothesis that assessments of PNI and extraprostatic invasion on a well characterized cohort with contemporary Grade group assessments, PSA and clinical stage added to the prognostic model and would help in decisions on active surveillance.

## RESULTS

**Figure 1:**
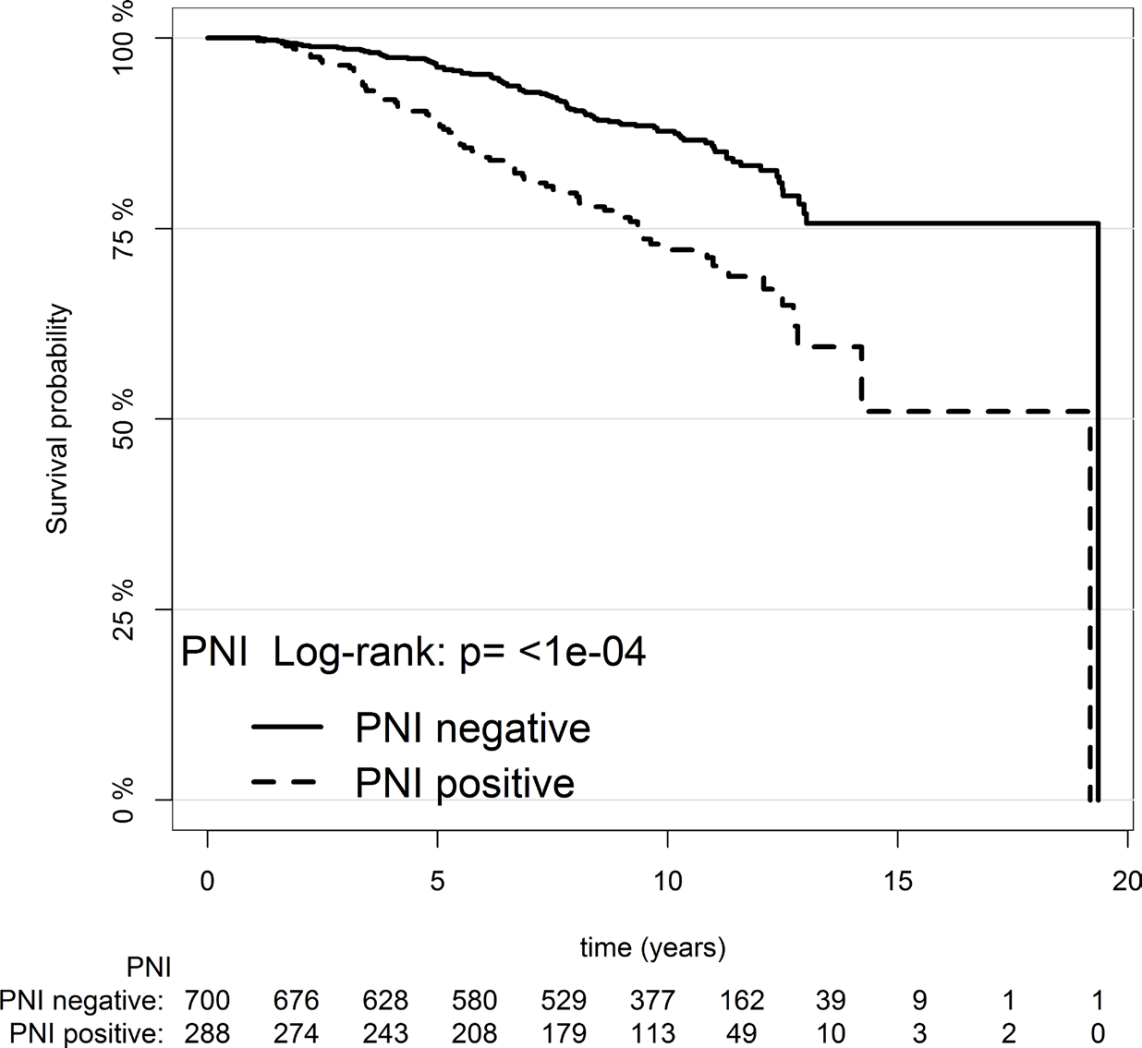
Kaplan–Meier survival estimate for patients with PNI versus patients without PNI

Of 988 patients, PNI was present in 288 (DOD = 75) and EPE in 23 (DOD = 5). Both were highly associated with GG (Pearson’s χ_1_^2^ *p*-value ≤ 2.2 × 10^–16^ and 5.1 × 10^–7^), respectively, and strongly associated with each other (Pearson’s χ_1_^2^ *p*-value = 2.8 × 10^–9^). Table 1 compares death from prostate cancer in the PNI positive and negative groups. On univariable analysis PNI was highly significantly associated with DOD (HR 2.28, 95% CI: 1.68, 3.1, log-rank test *p*-value = 4.77 × 10^–8^), but EPE was not (log-rank test *p*-value = 0.334). The median (interquartile range) for number of biopsies examined per case was 6 (8–5). Kaplan-Meier survival curves of PNI groups were plotted for the entire TAPG-needle patients (Figure 1).

**Figure 2:**
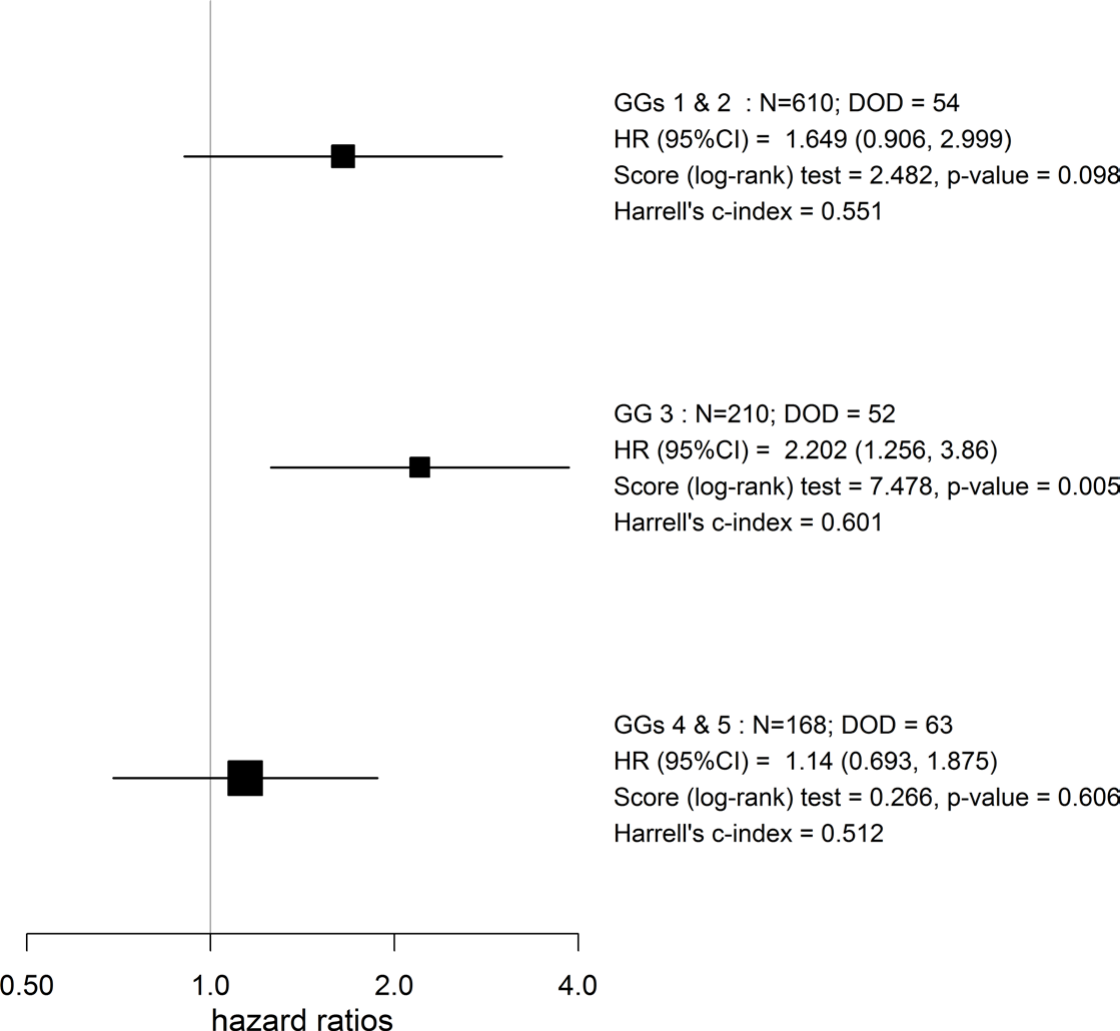
Forest plot of the PNI stratified by GG groups for time to DOD

Table 2 shows the results of univariable and multivariable statistical analyses of the cohort, by time to death from prostate cancer as outcome (Supplementary Table 1). On multivariable analysis with GG, serum PSA (per 10%), clinical stage and extent of disease (per 10%), PNI lost significance (HR 1.16, 95% CI: 0.83, 1.63, *p*-value = 0.371). However, PNI was the most informative predictor in GG 3 group (HR 2.20, 95% CI: 1.26, 3.86, log-rank test *p*-value = 0.005). However, PNI lost significance in combined GGs 1 and 2 as well as in combined GGs 4 and 5 (HR 1.65, 95% CI: 0.91, 3.0, log-rank test *p*-value = 0.098) and (HR 1.14, 95% CI: 0.69, 1.88, log-rank test *p*-value = 0.606) respectively (Figure 2).

**Table 2:** Summary of statistical analysis of TAPG-needle cohort, by death from prostate cancer (univariable and multivariable Cox models); Harrell’s c-index (95% CI) = 0.768 (0.722, 0.815)

		Univariable			Multivariable	
Predictor	*N* (*N*-event)	Hazard ratio (95% CI)	likelihood ratio χ^2^ (df, *P*)	c-index	Hazard ratio (95% CI)	likelihood ratio χ^2^ (*P*)^*^
Grade group	988 (169)		110.116 (4, <2 × 10^–16^)	0.732		110.116 (4, <2 × 10^–16^)
1	307 (15)	1 (reference)			1 (reference)	
2	303 (39)	2.81 (1.55, 5.10)			1.96 (1.05, 3.66)	
3	210 (52)	6.05 (3.40, 10.76)			3.34 (1.78, 6.28)	
4	56 (15)	7.12 (3.48, 14.57)			4.09 (1.93, 8.70)	
5	112 (48)	12.67 (7.09, 22.64)			5.16 (2.63, 10.12)	
PSA (per 10%)	988 (169)	1.24 (1.18, 1.31)	51.827 (1, 6.1 × 10^–13^)	0.684	1.08 (1.01, 1.15)	13.844 (1, 0.0002)
% disease (per 10%)	988 (169)	1.25 (1.19, 1.32)	78.437 (1, <2 × 10^–16^)	0.704	1.08 (1.01, 1.15)	10.020 (1, 0.0015)
T-stage	988 (169)		58.487 (3, 1.24 × 10^–12^)	0.650		8.599 (3, 0.035)
Stage 1	136 (15)	1 (reference)			1 (reference)	
Stage2	476 (54)	1.46 (0.81, 2.64)			1.04 (0.57, 1.89)	
Stage 3–4	146 (55)	5.76 (3.18, 10.41)			1.87 (0.99, 3.55)	
Stage-not recorded	230 (45)	2.07 (1.13, 3.78)			1.29 (0.69, 2.40)	
PNI			26.676 (1, 2.4 × 10^–07^)	0.601		
PNI-negative	700 (94)	1 (reference)			1 (reference)	
PNI-positive	288 (75)	2.28 (1.68, 3.10)			1.16 (0.83, 1.63)	0.802 (1, 0.371)
Age (years)	988 (169)	1.03 (0.997, 1.06)	3.165 (1, 0.075)	0.527		
ECE			0.805 (1, 0.370)	0.505		
ECE-negative	965 (164)	1 (reference)				
ECE-positive	23 (5)	1.55 (0.63, 3.76)				
					LR X^2^ = 143.380 (d.f. = 10, *p* < 2 × 10^–16^)	

^*^Terms added sequentially (first to last).

## DISCUSSION

A systematic review [4] in 2007 was conducted to examine the utility of reporting PNI in prostate cancer specimens. This found that study design, execution, and reporting precluded meta-analysis and quantitative risk estimation. Since then a large number of papers have been published on PNI, but very few which use prostate cancer death as the outcome. Many studies, even those in the recent literature, use pathological factors as surrogates for outcome [5–8]. Others use progression usually defined by biochemical recurrence [9–11] or metastasis [12].

Saeter *et al.* [13] demonstrated that the prognostic effect of PNI is dependent on an association with high grade carcinoma and reactive stroma. Most of the available studies are on patients treated by radical prostatectomy [14–19], some of which use preoperative biopsy data while other use pathological data from the radical prostatectomy specimen. Two studies used prostatic biopsy to predict death after external beam radiotherapy [20, 21]. To our knowledge only three previous studies have examined PNI in patients treated conservatively.

Moreira *et al.* [22] investigated 302 patients treated by active surveillance using disease progression as a surrogate for outcome. They showed that PNI was associated with a 73% chance of clinical progression after 2 years. Cohn *et al.* [23] examined 165 men similarly showed PNI predicted progression in both univariate and multivariate analysis. In neither case was survival data available.

Zareba *et al.* [24] investigated 615 men who underwent watchful waiting as part of the Swedish watchful waiting cohort. These men were diagnosed by trans-urethral resection of the prostate and not by biopsy. They showed that although PNI was significant in univariable analysis, this was not significant when adjusted for Gleason grade and tumor volume.

The studies examining PNI and prostate cancer death are summarized in Table 3. Seven out of 12 of these studies has failed to show any significance of the identification of PNI on multivariate analysis.

**Table 3:** Summary of studies which have examined perineural invasion as a prognostic factor in localized prostate cancer with prostate cancer death as the primary outcome

Author	Sample Type	Study size	Follow up (years)	Treatment	Univariable significance of PNI	Multivariable significance of PNI
Saeter *et al.*	Biopsy	318	10	Any	Yes	No
Tollefson *et al.*	Biopsy	451	12.9	RP	Yes	Yes
DeLancey *et al.*	Biopsy	3226	NR	RP	Yes	Yes
Feng *et al.* 2011	Biopsy	651	5.2	EBRT	Yes	Yes
Beard *et al.* 2004	Biopsy	517	4.5	EBRT	Yes	No
Aaltomaa *et al.* 2006	RP	211	NR	RP	Yes	No
Andersen *et al.*	RP	535	7.4	RP	Yes	Yes
Lee *et al.*	RP	361	3.5	RP	No	No
Van den Ouden	RP	273	4.1	RP	No	No
Zareba *et al.* (a)	TURP	615	9	WW	Yes	No
Zareba *et al.* (b)	RP	849	23	RP	Yes	Yes
Parameshwaran *et al.*	Biopsy	988	9.52	WW/Hormones	Yes	No

The challenges in looking for PNI are many, especially on biopsy specimens. Firstly, there may be no nerves for assessment in the biopsy. Only a relatively superficial biopsy of the prostate will contain nerves. The presence or absence of nerves in a biopsy is not regularly recorded, due to the difficulty in identification and laborious nature of this process. Therefore a ‘negative’ result in a biopsy containing prostate cancer may either indicate that nerves were present without invasion, or that no nerves were identified. This could be facilitated by immunochemistry [25], but would be far too expensive and time consuming to make it a viable proposition. It is interesting to note that PNI may be more easily observed by multiparametric guided MRI than by non-guided biopsies [26]. This may make assessment of PNI for active surveillance more of a viable proposition prospectively.

Examination for PNI itself is also very time consuming and in most busy pathology practices, it would seem to be wasteful of pathological time to examine every case for PNI where adequate risk stratification has already occurred.

We have shown, in keeping with other studies that PNI is a univariable risk factor for prostate cancer death, and have shown this is seen in a biopsy cases where the amount of material examined is far less than radical prostatectomy or TURP specimens. However the strength of this association was much attenuated in multivariable analysis with other standard clinic-pathological variables. In practical terms, the main method by which the identification of PNI might be clinically helpful beyond providing prognostic information, is in setting the criteria for active surveillance. Pathology plays a central role in terms of defining eligibility [27].

One study, dealt with the significance of PNI on needle biopsy in patients who are candidates for AS [28] but pathological surrogates for outcome were used. Cases with PNI had significantly greater likelihood of having more than 2 positive cores but showed no significant difference in surgical margin involvement or T3 disease at later radical prostatectomy or organ-confined disease. We therefore suggest that PNI should not currently be used to exclude from active surveillance protocols.

It has been suggested that extra-prostatic extension can be assessed on some prostate biopsies where the margins of the prostate have been sampled and invasion of fat assessed [29]. More recent studies have shown the presence of rare small fat foci within the prostate often mixed with benign glands [30, 31]. However in most cases it should be possible to distinguish extraprostatic fat from foci within the prostate to perineural invasion. As EPE was identified on only on 29/988 cases (2.9%) this study was not well powered and failed to reach significance. However in prognostic terms identification of EPE appears not to be important in this series. We therefore suggest it remain an optional item for reporting in pathological datasets. The strengths of this study include the large cohort size, the use of outcome data and the centralized nature of the pathology review. Weaknesses include the nature of the cohort data from routine medical notes and the fact that while conservatively treated, the patients were not treated to current standards of imaging or active surveillance protocols.

We suggest that examining prostate biopsy specimens for PNI is probably unnecessary, and that PNI remain a recommended rather than required part of national and International datasets. Its use as a potential exclusion criterion in patients entered for active surveillance is questionable and prospective studies using modern active surveillance strategies in conjunction with modern imaging are required for validation.

## MATERIALS AND METHODS

### Patients

Patient selection and data collection has been described in Cuzick *et al.*, 2015 [32]. In short, prostate cancer cases were identified from three cancer registries in Great Britain. Case notes were reviewed within collaborating hospitals. Patients were included in the study if they were diagnosed by needle biopsy at age less than 76 and had clinically localized prostate cancer between 1990 and 2003 inclusively. Patients were excluded if they were treated with either radical prostatectomy or radiation therapy within 6 months of diagnosis. Patients were also excluded if they had either objective evidence of metastatic disease (as detected by pelvic lymph node dissection, lymph node biopsy, bone biopsy, MRI, CT scan, radiograph or bone scan), or clinical evidence of metastatic disease (such as bone pain, spinal cord compression, soft tissue metastases, or pathological fracture). Other exclusion criteria included a PSA measurement of >100 ng ml^–1^ at or within 6 months of diagnosis, hormone therapy given prior to diagnostic biopsy, men who died within 6 months of diagnosis and men who had less than 6 months of follow-up. The study median follow-up time was 9.66 years with IQR 11.320–6.899.

Tissue specimens from the original needle biopsy were obtained and reviewed centrally by three specialist uropathologists to confirm diagnosis of adenocarcinoma and reassign Gleason score and Grade Group (GG). Patients were followed up to the 31st of December, 2012 through the cancer registries. Deaths were defined as either those due to prostate cancer (DOD) and those due to other causes by registry staff using death certificates only, in keeping with World Health Organization standardized criteria. The Northern Multicenter Research Ethics Committee provided national ethical approval, followed by local ethics committee approval at each collaborating hospital.

### Statistical method

Univariable and multivariable analysis was performed using a Cox proportional hazards model with the primary end point of analysis defined as death from prostate cancer. Observations stopped either on the date of death from other causes (DOC) or on the date of last follow-up. Co-variables included in the statistical analysis were GG, baseline PSA value (per 10%), clinical tumor T stage, % of tumor disease (expressed as cancer-positive cores out of total number of cores), and presence of PNI and ECE. Grade group analysis on this cohort has also been previously published [33]. Statistical analyses were performed using R [34].

There were three missing PSA values which were imputed using a median regression with patient’s age as a predictor and PSA as an outcome. All statistical tests were two-sided. No adjustment for multiple comparisons was made. *P*-values and 95% CI were based on likelihood ratio χ^2^ statistic with one degree of freedom obtained from partial likelihoods of proportional hazard models unless stated otherwise.

## SUPPLEMENTARY MATERIALS TABLE



## Abbreviations

CI: confidence interval
DOD: prostate cancer death (death of disease)
DOC: death from other causes
ECE: prostatic extension
GG: grade group
HR: hazard ratio
IQR: interquartile range
PC: prostate cancer
PNI: perineural invasion
PSA: prostate specific antigen
UK: United Kingdom
WHO: World Health Organization
