# Supplementary material for: Should reporting of peri-neural invasion and extra prostatic extension be mandatory in prostate cancer biopsies? correlation with outcome in biopsy cases treated conservatively
Source: Oncotarget. 2018 Apr 17;9(29):20555–62. doi: 10.18632/oncotarget.24994 (PMC5945501; doi:10.18632/oncotarget.24994)
Supplement: Supplementary file 1 [file oncotarget-09-20555-s001.pdf]

Should reporting of peri-neural invasion and extra prostatic extension be mandatory in prostate cancer biopsies? correlation with outcome in biopsy cases treated conservatively

SUPPLEMENTARY MATERIALS

Supplementary Table 1: Summary of statistical analysis of TAPG-needle cohort, by death from prostate cancer (univariable and multivariable Cox models)

		Univariable			Multivariable	
**Predictor**	***N* (*N*-event)**	**Hazard Ratio (95% CI)**	**likelihood ratio χ^2 (df, *P*)**	**c-index**	**Hazard Ratio (95% CI)**	**likelihood ratio χ^2 (*P*)***
Grade group	988 (169)		110.116 (4, <2 $\times 10^{-16}$)	0.732		110.116 (4, <2 $\times 10^{-16}$)
1	307 (15)	1 (reference)			1 (reference)	
2	303 (39)	2.81 (1.55, 5.10)			1.94 (1.04, 3.63)	
3	210 (52)	6.05 (3.40, 10.76)			3.31 (1.76, 6.23)	
4	56 (15)	7.12 (3.48, 14.57)			3.99 (1.87, 8.51)	
5	112 (48)	12.67 (7.09, 22.64)			5.14 (2.62, 10.07)	
PSA (per 10%)	988 (169)	1.24 (1.18, 1.31)	51.827 (1, 6.1 $\times 10^{-13}$)	0.684	1.08 (1.01, 1.15)	13.844 (1, 0.0002)
% disease (per 10%)	988 (169)	1.25 (1.19, 1.32)	78.437 (1, <2 $\times 10^{-16}$)	0.704	1.08 (1.01, 1.15)	10.020 (1, 0.0015)
T-stage	988 (169)		58.487 (3, 1.24 $\times 10^{-12}$)	0.650		8.599 (3, 0.035)
Stage 1	136 (15)	1 (reference)			1 (reference)	
Stage2	476 (54)	1.46 (0.81, 2.64)			1.03 (0.57, 1.88)	
Stage 3–4	146 (55)	5.76 (3.18, 10.41)			1.86 (0.98, 3.53)	
Stage-not recorded	230 (45)	2.07 (1.13, 3.78)			1.28 (0.69, 2.39)	
PNI			26.676 (1, 2.4 $\times 10^{-07}$)	0.601		
PNI-negative	700 (94)	1 (reference)			1 (reference)	
PNI-positive	288 (75)	2.28 (1.68, 3.10)			1.16 (0.83, 1.62)	0.802 (1, 0.371)
Age (years)	988 (169)	1.03 (0.997, 1.06)	3.165 (1, 0.075)	0.527	1.01 (0.98, 1.04)	0.343 (1, 0.558)
ECE			0.805 (1, 0.370)	0.505		
ECE-negative	965 (164)	1 (reference)				
ECE-positive	23 (5)	1.55 (0.63, 3.76)				
					LR X^2 = 143.723 (d.f. = 10, $p < 2 \times 10^{-16}$)	
					Harrell's c-index = 0.769 (se = 0.024)	
					N = 988; *n*-event = 169	

* Terms added sequentially (first to last).
